# Multi-Sensor Fault Detection, Identification, Isolation and Health Forecasting for Autonomous Vehicles

**DOI:** 10.3390/s21072547

**Published:** 2021-04-05

**Authors:** Saeid Safavi, Mohammad Amin Safavi, Hossein Hamid, Saber Fallah

**Affiliations:** 1 Department of Mechanical Engineering Sciences, Connected Autonomous Vehicle Lab (CAV-Lab), University of Surrey, Guildford GU2 7XH, UK; s.safavi@surrey.ac.uk (S.S.); h.hamid@surrey.ac.uk (H.H.); 2 Department of Mechanical Engineering, Isfahan University of Technology, Iran 84156-83111, Iran; safavi.m.amin@me.iut.ac.ir

**Keywords:** health forecasting, fault prediction, fault detection, fault isolation, machine learning

## Abstract

The primary focus of autonomous driving research is to improve driving accuracy and reliability. While great progress has been made, state-of-the-art algorithms still fail at times and some of these failures are due to the faults in sensors. Such failures may have fatal consequences. It therefore is important that automated cars foresee problems ahead as early as possible. By using real-world data and artificial injection of different types of sensor faults to the healthy signals, data models can be trained using machine learning techniques. This paper proposes a novel fault detection, isolation, identification and prediction (based on detection) architecture for multi-fault in multi-sensor systems, such as autonomous vehicles.Our detection, identification and isolation platform uses two distinct and efficient deep neural network architectures and obtained very impressive performance. Utilizing the sensor fault detection system’s output, we then introduce our health index measure and use it to train the health index forecasting network.

## 1. Introduction

Autonomous vehicles are already roaming modern cities’ streets thanks to the state-of-the-art machine learning algorithms and controllers. These methods are heavily dependent on data from multiple sensors. Due to this reliance, sensor fault and the resulting error propagation can be detrimental to the reliability and safety of autonomous vehicles, giving importance to sensor health monitoring and prognostics. The purpose of a sensor health monitoring system is to not only detect, isolate and identify the faulty sensors but also try to predict the performance and reliability of sensors and make decisions based on the prediction [1].

Fault is characterized as an unauthorized deviation from the normal state of at least one characteristic property or parameter of the system [2]. Faults can be categorized as sensor faults, actuator faults and part or process faults [3]. Sensor faults point to the faults in the input module, and the actuator faults address the faults in the output module. To eliminate any potential danger and safety risk, vehicles with a very complicated electronic architecture require an appropriate fault diagnosis strategy [4] including fault detection which shows if there is fault present in the system, fault isolation that is used to find which sensor is faulty, fault identification that determines how the sensor has failed and sensor health forecasting strategy which shows the current and future state of the sensors.

Different methods and techniques have been proposed by researchers to detect/identify the nature of faults and isolate the defective sensor. For instance, a data-driven approach was suggested by [5] named improved credit assignment cerebellar model articulation controllers neural network knowledge fusion model which included fault data preparation, fault detection and isolation of controllers. By using sensor, actuator data and motion state value, Ref. [6] developed an intelligent decision subsystem, understanding the risk analysis of autonomous robots. Focused on the SVM, Ref. [7] found and successfully determined and isolated the autonomous vehicle’s sensor fault. In addition, the efficacy of learning techniques in fault identification and isolation has been demonstrated in the aircraft industry [8]. These previous work bodies suggest that the application of the fault detection, identification and isolation of autonomous vehicles to the field of learning techniques will theoretically provide great benefits in terms of vehicle safety.

For sensor health monitoring and fault diagnosis, initially model-based and rule-based models were used; however, deriving theoretical models and rules for complex systems can be a complicated task. With the recent growth of computational power, memory capacity and sensor technology, data-driven methods like machine learning and statistical models gained more attention and proved to be more effective [9]. Currently, deep learning methods have been in the center of attention as the state-of-the-art frameworks [10]. These methods have been applied to some health monitoring approaches based on different assumptions and data-availability. In general, the methods can be categorized as [11]: (i) knowledge-based, such as model, rule and ontology-based methods; (ii) statistical methods, such as traditional machine learning methods, e.g., support vector machines (SVMs), K-nearest neighbors (K-NN) and artificial neural networks (ANNs) and (iii) deep learning methods, such as deep neural networks (DNNs), recurrent neural networks (RNNs), convolutional neural networks (CNNs), autoencoders (AE) and estricted Boltzmann machine (RBM).

Among the traditional machine learning methods, SVMs and random forests are the common techniques that are used for system health monitoring and they act as the golden standard. The main advantage of these approaches is their excellent generalization capabilities, resulting in high classification and regression accuracy for condition monitoring and diagnosis [11]. However, most of these methods require hand-crafted features that can consistently capture faults. Additionally, the majority of statistical methods suffer from curse of dimensionality and can be very computationally expensive if multiple data-streams are involved. These shortcomings have been the main motivations to use deep learning methods for feature extraction and selection, and dimension reduction [10]. Table 1 provides a comprehensive review of the various deep learning architectures which have been utilized for health management systems and their strengths and limitations.

**Figure 1:**
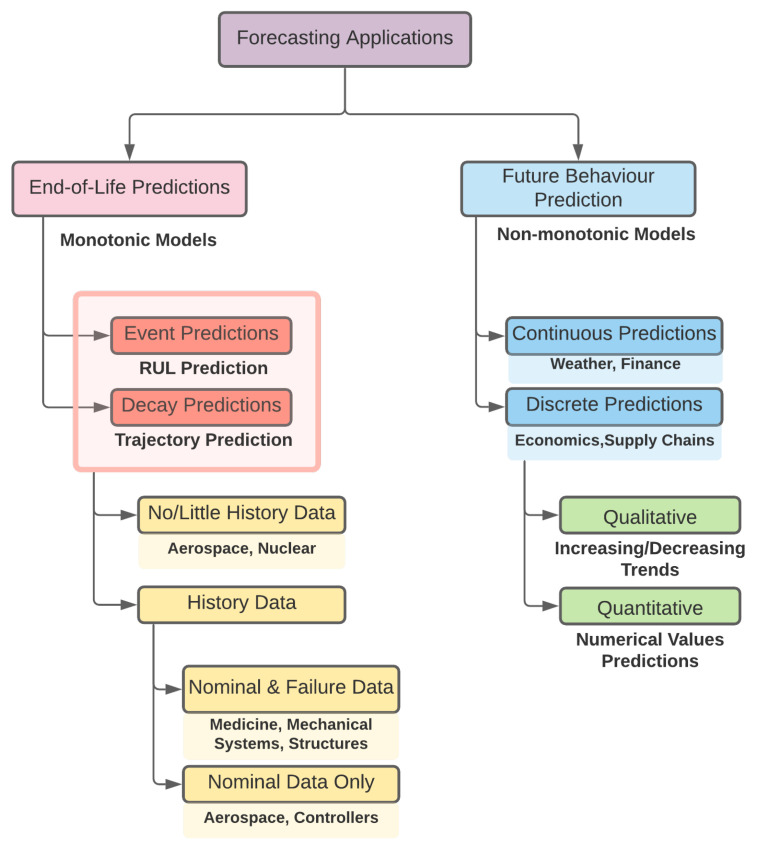
Different forecasting categories for various applications [12].

Health prognostics is often viewed as an application of forecasting. Forecasting applications can be categorized in two major classes, as shown as in Figure 1. The majority of the research in prognostics and health management (PHM) has been dedicated to End-of-Life predictions, and these applications of the studied component, such as turbines, batteries, gears, bearings, etc., have a certain critical threshold of failure and system’s health is considered to be monotonic [12].

In the context of health prognostics, methods can be grouped as [13]: (i) remaining useful life (RUL) prediction using regression models, (ii) early failure detection using classification techniques and (iii) anomaly detection, analysis and prediction based on detection.

For RUL prediction, the central assumption is that historical run-to-fail data of the system are available. This is mostly possible for analyzing the health status of mechanical components, e.g., turbofan engine [14] and rotary machinery [15], in which degradation data are available from accelerated degradation tests [13]. However, these valuable datasets are only available for a limited selection of mechanical components, and little data are available on the degradation of electronic sensors. The RUL prediction algorithms learn the degradation path of the studied component.

The main goal of early failure detection is to identify failures by learning the sequential patterns of data that lead to failure from historical data. This can be represented as a binary classification problem. Signals which will follow a failure are labeled as faulty, and others are labeled as the healthy class. The downside of this approach is that the system can only foresee a short time window in the future and can not forecast long-term status; that is why these methods are called early failure detection.

The main focus of this paper for health monitoring strategy is on anomaly detection, analysis and prediction. This prediction is based on the output of the detection platform. This approach, also known as outlier detection, is traditionally applied to fraud detection, target tracking, personal health monitoring and network security breaches and is currently being extended to the area of wireless sensor networks. In this context, sensor outliers are considered as irregularities in observations that are not consistent with the pattern of behavior given the circumstances of a scenario. However, there is no universal definition of sensor outliers in the literature [16]. Table 2 provides an all-inclusive selection of definitions used in the literature.

One of the challenges in sensor prognostics is detecting context-related anomalies in the sensor outputs effectively and making predictions based on the detection results. However, it is known from the anomaly detection scenarios that abnormal observations are scarce compared to the sensors’ normal behavior, which results in considerable imbalanced data. The other challenge is a lack of ground truth on the degradation path of sensors. This challenge is due to the fact that the run-to-failure data on sensors, which are generally available for RUL of mechanical parts, are not available for electronic sensors.

Considering the gaps in multi-sensor, multi-fault detection and identification, and condition-based fault prediction, this paper proposes a novel multi-sensor, multi-fault resilient architecture to meet the current research gaps. The present research gaps that we have addressed in this research are the following. The first is providing a reliable health index measure for an unbalanced dataset of healthy and faulty classes. The second contribution is implementing a novel predictive algorithm that forecasts sensors’ health index, which can estimate the time at which degrading performance of a sensor would cause a critical failure. These forecasts can be used for sensor network prognostics and aid an accurate maintenance schedule. Moreover, the prediction algorithm provides interpretability and risk management measures using attention mechanisms and quantile regressions. These considerations for practical implementations would represent a step forward in addressing the current research gaps for bringing fault detection, isolation/identification and prediction into autonomous vehicle applications. The third contribution of this research is designing a unified framework and methodology for reliable fault detection, identification and prediction scalable for multiple sensors. Towards this end, a novel method is proposed for predicting the health status of the electronic sensors of autonomous vehicles. A CNN classifier has been used for real-time multi-sensor monitoring to capture faulty signals and construct a health index (HI) for sensors. The evolution of the HI is then used to forecast the sensor’s health and possible depredations. This process is represented as a multi-step uni-variate time series forecasting problem using a Temporal Fusion Transformers (TFT) network. We have also proposed a feature extraction and DNN classification technique to isolate and identify the detected fault type. To test our method, we have used the recently released real-world dataset from a car manufacturer (Audi) [21].

The structure of this paper is as follows: In Section 2 the dataset and the partitions which were used for our experiments are described. Section 3 contains the system description for multi-sensor fault detection, identification/isolation and prediction. Subsequently, Section 4 summarizes the obtained results and their corresponding analysis. Finally, Section 5 concludes the findings of this research.

## 2. Dataset Description

Audi recently released an autonomous driving dataset (A2D2) [21], which includes data derived from the automobile bus and recorded on highways, country roads and cities in the south of Germany. Vehicle bus data are stored in a JSON-file, which contains the bus signals themselves as well as the corresponding timestamps and units. The signals comprise odometer, acceleration, (angular) velocity, GPS coordinates, brake pressure, pitch and roll angles. By including vehicle bus data, Audi not only allows A2D2 to be used for imitation learning research but also enables reinforcement learning approaches.

**Figure 2:**
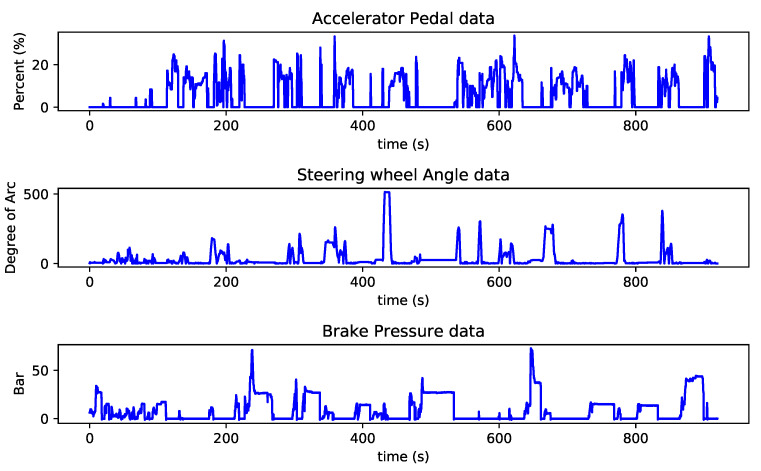
Three vehicle bus data-points are depicted over time. The top figure shows the output of the Accelerator Pedal (AccP) sensor, the middle figure is for the Steering Wheel Angle (SWA) and the bottom figure is showing the output of the Brake Pressure (BP) sensor.

In this paper, we have used urban and highway driving scenarios from the A2D2 dataset using the Audi bus controller, which came from three sensors’ output. These sensors are Accelerator Pedal (AccP), Steering Wheel Angle (SWA) and Brake Pressure (BP). We have used all available data from three sensors, approximately 35 min of driving in three cities in total. These outputs (sequential data-points) are then converted to signals with a length of 2.4 s (the sensors’ sampling frequency is 100 Hz, which means 10 ms period). In Figure 2, there is an example of output from three sensors (AccP, SWA and BP) over time, which was recorded from driving sessions at Ingolstadt.

Table 3 shows the available real data-points from three sensors and per recording location for the A2D2 dataset.

In order to have a coherent open-set of experiments throughout the paper, we have defined the fixed methodology for using the available data. As explained earlier in this section, the data were recorded from three different cities. We have used all available data from Ingolstadt, Gaimersheim and Munich for training, validation and testing, respectively. Ingolstadt has the highest number of samples, so we have used data from this city to train our networks. Gaimersheim has the lowest number of data, and it has been chosen for validation and parameter optimization. By keeping the data constant for all the experiments, we can first be sure that none of the data in validation and testing phases are visible to the network during training (means open-set scenario). Secondly, since our prediction architecture is based on our detection network’s output, we can be sure that no underlying information is passed to the prediction network from the detection phase.

### Fault Injection for Fault Detection Task

Sensor high reliability assurance necessitates a thorough understanding of their failure modes. Sensor failures may be caused by a variety of factors, including aging, wear, manufacturing deficiencies, incorrect calibration or mishandling and external conditions [22]. For instance, electromagnetic interference can cause sensors to become inactive or noisy, or corrosion can degrade PCBs. As it can be seen, some of the causes of faults and failure are temporary, such as electromagnetic and insulation disturbances [23]. As a consequence, sensors’ health can fluctuate during its life span. This implies that a monotonic health index, which is commonly used for mechanical components, can not be used effectively in this context. For this reason, we have introduced a non-monotonic health index in Section 3.3.1 that can be used to forecast the future health state of the sensor network.

A number of different fault patterns may occur in a system, such as total sensor failure, intermittent or continuous faults. The intermittent faults refer to the faults that occasionally happen, while the continuous faults refer to those that persist in the system. The intermittent or continuous faults which we have considered are classified as follows [23,24,25]:

- Drift fault refers to the situation where a signal slowly deviates (linearly) from the actual value.
- Hard-over fault refers to the situation when a sensor returns a value outside its measurable range and increases to the saturation point for a short time.
- Erratic fault occurs when the sensor data have a large increase in noise, as a result, the magnitude of the signal variance can increase around the true value over time.
- Spike fault happens when occasional increases in the signal value are produced. The density of spike faults within the signal can increase over time.

To create faulty signals, we have injected four different types of faults to the healthy signals from three sensors (AccP, SWA and BP) of the A2D2 dataset described in Section 2. There are two main benefits of using this methodology [17]. Firstly, adding faults into sensor measurements provides an accurate “ground truth” which allows one to better understand the efficacy of a detection algorithm. Secondly, fault intensity and frequency can be changed for different scenarios, allowing us to test the limits of each method and comparing various schemes at low fault intensities. Despite the fact that faults and failure thresholds that we have found in existing literature are of relatively high intensity (a considerable fraction of full scale output), we believe it is critical to understand the behavior of fault detection methods over a spectrum of fault intensities, as it is uncertain if the failure threshold would be as prevalent for different sensor systems and state-estimation methods used for autonomous vehicles. The injection of each fault incorporates pseudo-randomness to simulate the variability of each fault condition. Intermittency of fault conditions has been included for hard-over, erratic and spike to simulate real-world fault conditions. We have injected faults to all the available data from three cities to provide sufficient training material of different scenarios.

The method used to inject the fault to the A2D2 dataset is designed to cover all possible combinations of faults in three channels (each channel contains a stream of signals from AccP, SWA and BP sensors) and also fault types. Table 4 shows the combinations which we have used to generate faulty signals. In Table 4, F and H represent faulty and healthy signals. For faulty signals, we have injected and generated all four types of faults for each combination. The dataset is accessible from the Github repository via this link. (https://github.com/MAminSFV/multi-sensor-FDII-health-forecasting-for-autonomous-vehicles (accessed on 3 April 2021))

The fault is introduced after the 25th percentile of one or more of the three sensor signals. As shown in Table 4, the type and location (which sensor is faulty) of fault are introduced to cover all possible combinations, so the machine can learn more by observing more. Normal distributions have been used to simulate the amplitude of faults where applicable. An error of 20% of Full-Scale Output (FSO) has been defined as faulty for the erratic and drift faults.

**Figure 3:**
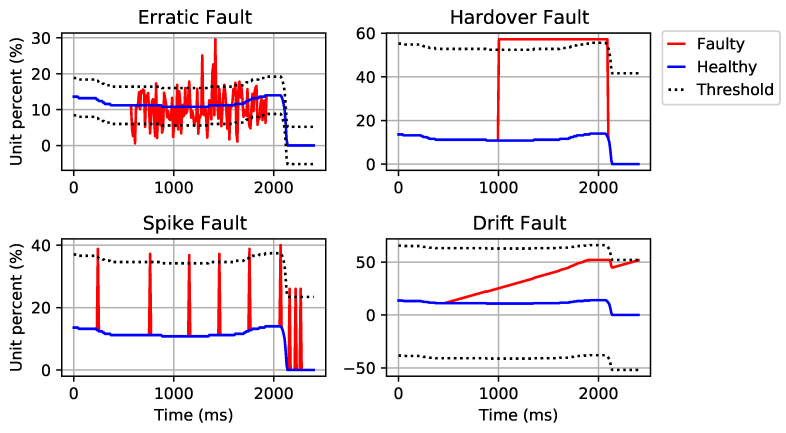
Examples of sensor faults. Data have been taken from the Audi autonomous driving dataset (A2D2). All four figures are faulty variants of a sample signal from the AccP sensor.

The fault parameters shown in Table 5 have been selected to give a suitable representation of real-world faults for the simulated data. Uniform distributions are used to incorporate randomness into drift fault gradient, hard-over fault length and erratic fault length. The erratic fault amplitude uses a normal distribution with a standard deviation of 0.2. Examples of the described faults are shown in Figure 3.

## 3. System Description

The topic explored in this paper is broadly described as Integrated Vehicle Health Monitoring. The benefits of Integrated Vehicle Health Monitoring systems are improved vehicle safety, more accurate health awareness and reductions in through-life costs.

The proposed system in this paper comprises both current vehicle state awareness (fault detection and identification) and prediction to determine future vehicle health. An adequate understanding of the vehicles current health state would allow for intelligent vehicles to reconfigure the available healthy systems to compensate for failures, or for maintenance to be directed quickly to the faulty components. Predictive fault detection allows for the condition of the vehicle systems to be predicted at a future point in time. The proposed predictive system provides the opportunity to control systems to reconfigure themselves in advance of a sensor failure or fault.

**Figure 4:**
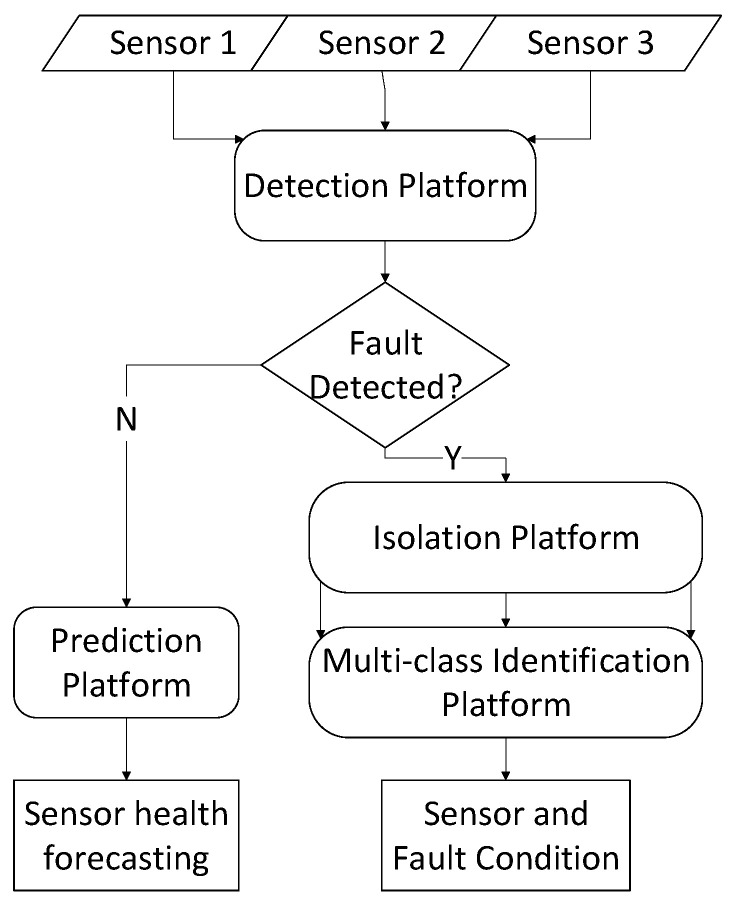
Proposed system architecture.

The overall architecture of the proposed system is shown in Figure 4 which consists of detection, isolation, identification and prediction modules. Each element of system architecture, shown in Figure 4, is described in the following subsections.

### 3.1. Sensor Fault Detection

It is important to identify sensor faults early on, and ANNs are commonly used for this task. Two separate blocks are generally used for typical systems: extraction of features and classification. These customized and hand-crafted features can be a sub-optimal solution and entail a high cost of computing, impeding their use for real-time applications. In this article, using 1D CNNs with an intrinsic adaptive architecture, we used a fast and precise sensor monitoring and early fault detection method to fuse the feature extraction and classification phases of the sensor fault detection into a single learning body. This approach is based on the proposed method by Ince et al. [26]. This is immediately effective for the raw data and alleviates the need for a distinct feature extraction process that results in a more effective method in terms of both speed and precision.

**Figure 5:**
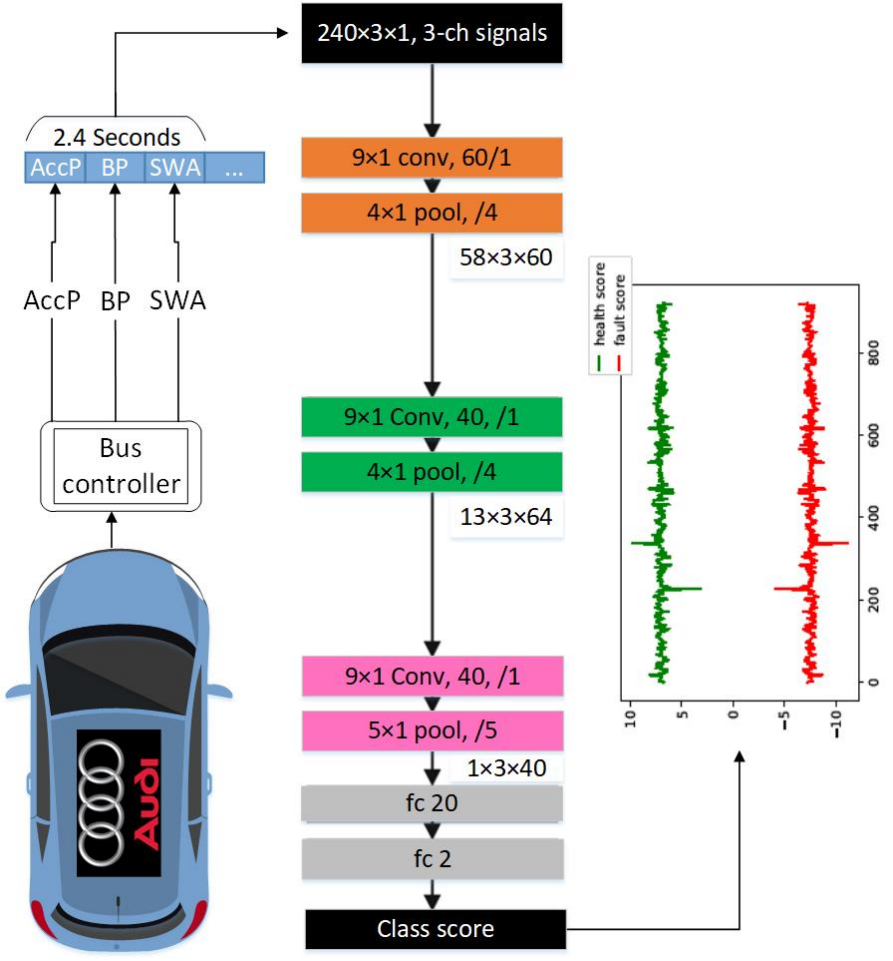
The convolution structures of the suggested 1D CNN adaptive configuration [26], e.g., “9×1 conv, 60, /1” indicates a convolutional layer with 60 kernels, size 9×1, stride 1; a box labeled “4×1 pool, /4” implies a max pooling layer with a size of 4×1, stride 4; ‘fc 20’ is a fully connected layer with the output dimension of 20. The numbers across boxes are the prior layers’ output sizes.

A CNN 1D architecture was used to fuse the extraction and learning processes of the raw sensor signals. The flexible CNN configuration will enable us to work with every input layer dimension. In addition, the suggested compact CNN has hidden convolution layer neurons that can execute both convolution and subsampling operations. The 1D CNNs are composed of an input layer, hidden CNN and fully connected layers and an output layer. The key structural distinction between conventional 2D and suggested 1D CNNs is the use of 1D kernels instead of 2D kernels. By this means, the number of weights and computation decreases and 2D matrix manipulations such as 2D convolution and lateral rotation have now been replaced by their 1D replacements, 1D convolution and reverse rotation [26]. The overall block diagram of the CNN architecture is illustrated in Figure 5. As shown in the figure, the three sensor signals are stacked as three channels and fed into the input layer. The length of the moving window is considered to be 2.4 s, as suggested by Ince et al. [26]. The CNN model is then structured hierarchically with convolutional layers, pooling layers and fully connected layers. Convolutional kernels can effectively capture relevant temporal input connections across the input channels. This network is able to detect faults by observing 2.4 s of the three data streams. Additionally, this model is computationally fast due to its compact architecture, using only three 1D convolutional layers.

### 3.2. Sensor Fault Identification and Isolation

The proposed architecture for identification and isolation consists of two parts: feature extraction and multi-class DNN modeling. For sensor fault identification and isolation tasks we have used a separate feature extractions block as the real-time implementation is not a concern (and it could be done offline) as opposed to the fault detection task which needs to be done in real time. DNNs are used to identify the type of fault in sensors and to recognize the faulty sensors. Typically, deploying these modules together allows the intelligent system to make state identifications and a decision based upon the current state for sensor isolation.

#### 3.2.1. Feature Extraction

As shown in Figure 4, once a fault is detected in the fault detection stage, the faulty and non-faulty signals are imported into the feature extraction function. In this paper, this step is done using a feature extraction algorithm which is applied to each signal. The available techniques for feature extraction in the literature can be classified as time-domain analysis, frequency-domain analysis and a mix of both [27].

Refs. [28,29] summarize 10 widely used time-domain features which can typically be generalized to a variety of applications. In the feature extraction module of this paper, 10 time-domain features, which are defined in Table 6, are used to generate a feature matrix, where each row includes 10 values of a sample by a length of 240 data points.

#### 3.2.2. Fault Isolation and Identification Based on Multi-Class DNN

**Figure 6:**
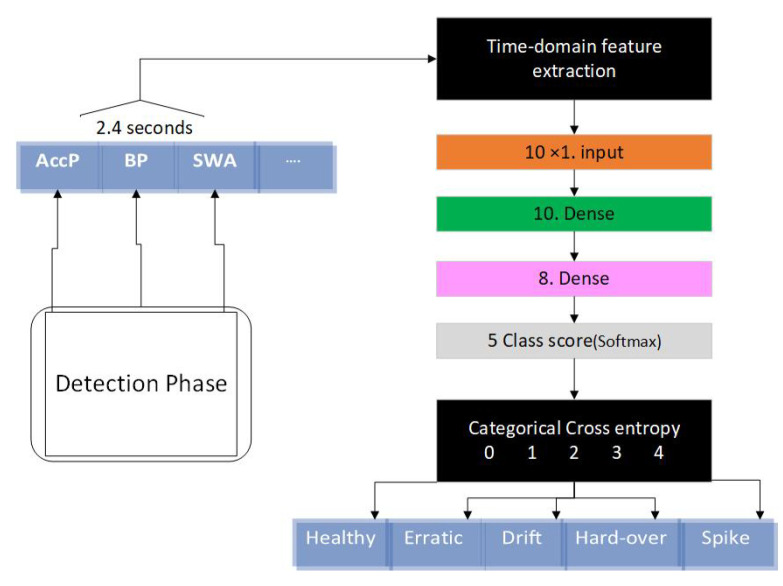
The proposed multi-class deep neural network (DNN) architecture in this study for fault isolation and identification.

DNN-based methods shown fast and high-accuracy performance in fault/anomaly identification [30]. In the feature extraction phase, 10 distinct features are extracted from the sensor signals. A normalization (in the range of 0 to 1) is applied to the extracted features. In the next step, the data are divided into training, validation and testing sets as described in Section 2. A four-layer DNN architecture, consisting of one input layer, two hidden layers and one output layer, has been developed for isolation and identification purposes (see Figure 6). The number of nodes in the input layer is equal to the size of the extracted features from the signals. Eight hidden layers are also used in our DNN architecture. The output layer uses a softmax function to develop four classes (for all fault types) and one class for the non-faulty signals, as presented in Figure 3.

Multi-class DNNs for fault identification and isolation are developed in a way to minimize the categorical cross-entropy loss function.

### 3.3. Sensor Health Forecasting

The health state forecasting platform needs a proper health index definition and forecasting strategy to learn different patterns of health index measures. In subsequent subsections, we have initially defined a novel health index measure and then our sensor health forecasting strategy which is based on the usage of a TFT network [31].

#### 3.3.1. Health Index Definition

Considering the fact that the A2D2 dataset does not include degraded signals, a degradation process needs to be introduced to each driving scenario. In this work, different fault patterns with three degradation paths have been artificially added to A2D2 dataset to make the learning process as general as possible. For example, continuous erratic, drift and spike faults with linear, exponential and sinusoidal amplitudes are used as a degradation scenario. These degradation paths are adopted based on the patterns studied in [22].

**Figure 7:**
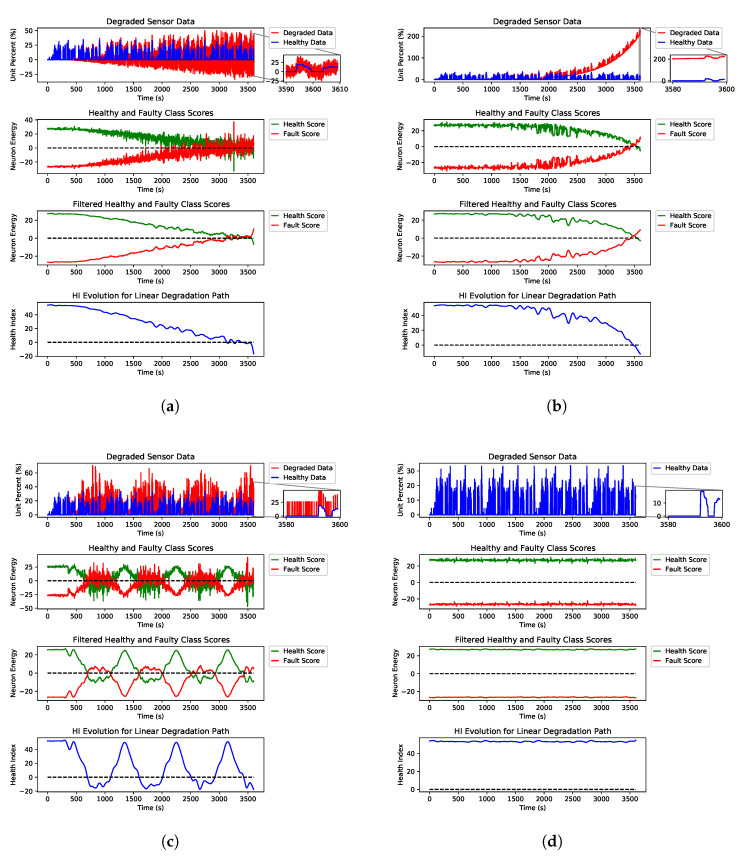
Three different degradation scenarios for erratic, drift and spike fault types of the AccP sensor. (**a**) Steps taken to construct the HI from a linearly degraded AccP data stream with erratic fault. (**b**) Steps taken to construct the HI from an exponentially degraded AccP data stream with drift fault. (**c**) Steps taken to construct the HI from a sinusoidal degraded AccP data stream with spike fault. (**d**) Steps taken to construct the HI from a healthy AccP data stream.

**Figure 8:**
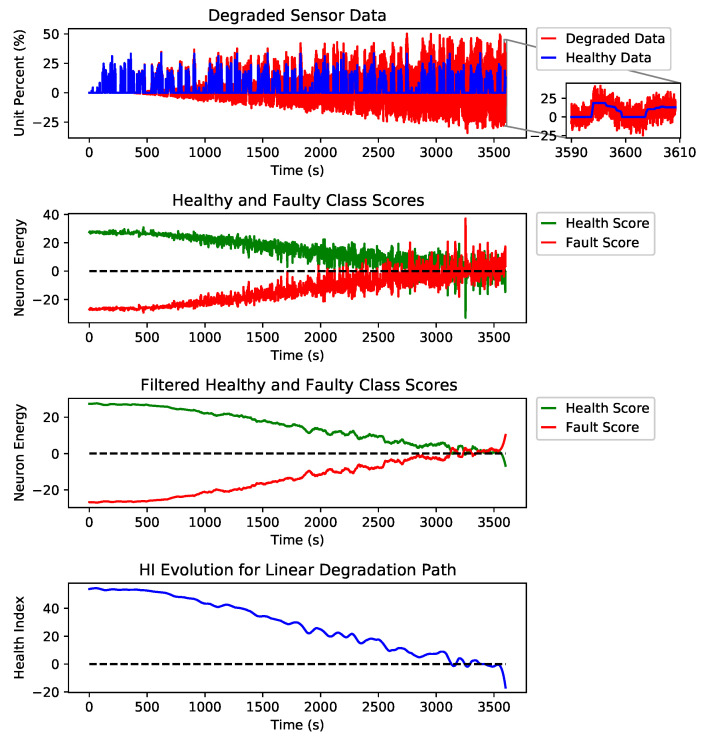
Steps taken to construct the HI from a linearly degraded AccP data stream.

**Figure 9:**
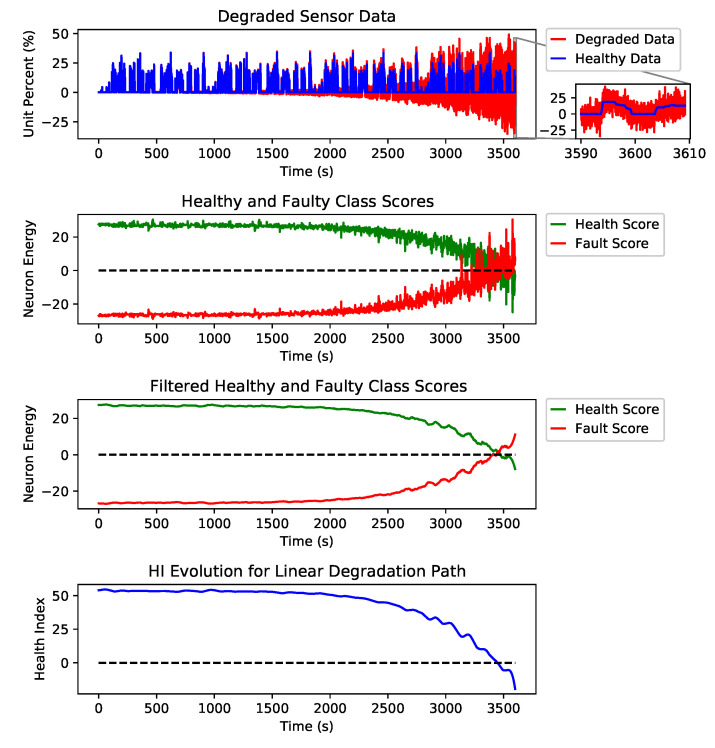
Steps taken to construct the health index (HI) from an exponentially degraded AccP data stream.

**Figure 10:**
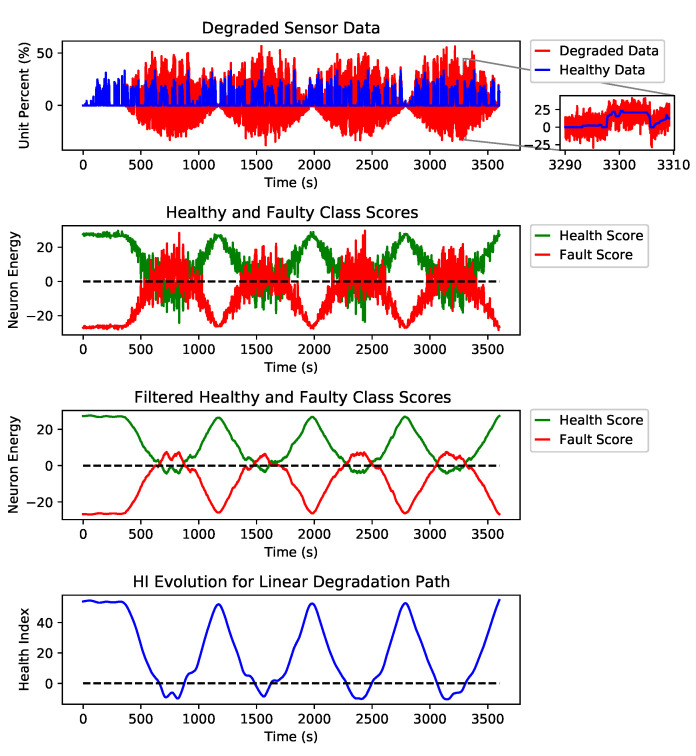
Steps taken to construct the HI from a sinusoidal degraded AccP data stream.

Figure 7 shows examples of three different degradation paths for fault types of erratic, spike and drift on the AccP sensor and the healthy signal with no faults. These are just examples to show how the HI was constructed from the output of our detection engine. Still, for prediction, we have trained our model using all three degradation paths and all fault types for all signals along with all healthy signals in the training split. Figure 8 shows the linear degradation of erratic fault for AccP signal, Figure 9 and Figure 10 demonstrate exponential degradation of drift fault and sinusoidal degradation of spike fault for AccP signal, respectively. Figure 7d shows the HI construction process for the healthy AccP signal.

The blue data-point sequence in the top figure represents the healthy, and the red data-point sequence shows the same sequence, which is degraded by fault over time. The second plot shows the CNN detector’s output when the fault is gradually added to the healthy signal. The third plot is the filtered version of the second plot, which shows the overall trend for the detection network’s output. The bottom plot is the subtraction of healthy scores (green) from the faulty scores (red) in the second plot. The HI is constructed based on the output of the fault detector network.

The last layer of the CNN architecture is equipped with a linear activation function against the conventional softmax and other activation functions used for the classification task. The main reason to choose a linear activation function is to have an unbounded and one to one map for each class score. One emergent property is that class scores increase linearly as fault intensity increases, as found in Figure 7. The network provides two scalar scores for each class, green and red plots are showing the healthy and faulty output of the detection network, respectively. These scalar outputs show the quality of the signal, and by monitoring the evolution of these scores through time, we will be able to detect possible degradation. However, these scores are quite noisy since a linear activation function was used. To find trend lines and meaningful patterns in the evolution of scores, a Savitzky–Golay filter is applied to de-noise the scores and extract smooth trend lines. The filtered scores are shown in the third plot of each subfigure. The HI is defined as the subtraction of the filtered scores. A healthy sensor system is expected to have a positive HI, and as the sensors’ quality degrades, the HI decreases, and a sensor failure happens when the HI sign is changed to negative.

For HI measure, we have tested our proposed method with three fault types; erratic fault, which has the most random characteristics, drift and spike. Hardover fault is not used for prediction because it happens suddenly and early fault detection is the best approach to prevent system failure in this case.

As explained in Section 2, the desired health index should be non-monotonic since electronic systems can recover if the external interference is removed. In addition to non-monotonicity, HI should be robust to noise and present a smooth degradation trend. As shown in Figure 7, the proposed HI demonstrates these features for different degradation scenarios.

The Y axis in the second and third plots of each sub figure are marked as neuron energy which means the output (this is the weighted sum of neuron inputs plus the bias term) of each corresponding neuron per class.

#### 3.3.2. Sensor Health Forecasting Strategy

For sensor health forecasting based on detection, we have trained the TFT network [31] using the HI obtained in the previous section. The HI measures are obtained from each of the three cities, and all possible fault combination described in Table 4 are considered. TFT was proposed by Lim et al. [31] in 2020 and is introduced for multi-horizon forecasting. Multi-horizon forecasting also involves a dynamic combination of variables, including static covariates, known future inputs and other exogenous time series, which are observed only in the past (with no prior information on how they link with the target). TFT is an attention-based structure that provides high-performance multi-horizon forecasting. TFT uses recurrent layers for local processing to learn temporal associations at various scales. TFT uses advanced components to pick appropriate features and a set of gating layers to suppress redundant components, allowing high performance in a wide variety of scenarios. This method performed impressively on various real-world datasets, and the authors demonstrated significant performance improvements over existing benchmarks.

**Figure 11:**
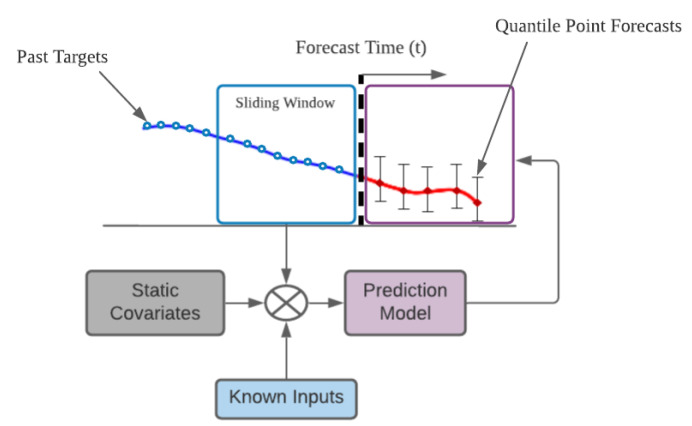
Sketch of static covariate multi-horizon forecasting and past-observed inputs.

Functional multi-horizon forecasting applications typically involve access to a number of sources of data, including static metadata, as seen in Figure 11 (e.g., city where the data are recorded).

The TFT is designed for using canonical components for a wide variety of problems to effectively construct feature representations for each input type (i.e., static, documented, observed inputs) for high forecasting efficiency. TFT’s main components are:

- Gating structures to skip over any redundant elements of the architecture, to support a wide variety of datasets and scenarios, offering adaptive depth and network complexity.
- Networks of variable selection to choose appropriate input variables at each stage of the time.
- Static covariate encoders to incorporate static features into network by encoding contextual vectors to condition temporal dynamics.
- Temporal computation from both observed and known time-varying signals to learn both long- and short-term temporal relationships. For local processing, a sequence-to-sequence layer is used, while long-term dependencies are collected using an innovative interpretable multi-head attention block.
- Quantile forecast prediction periods to assess the spectrum of likely target values for each prediction horizon.

**Figure 12:**
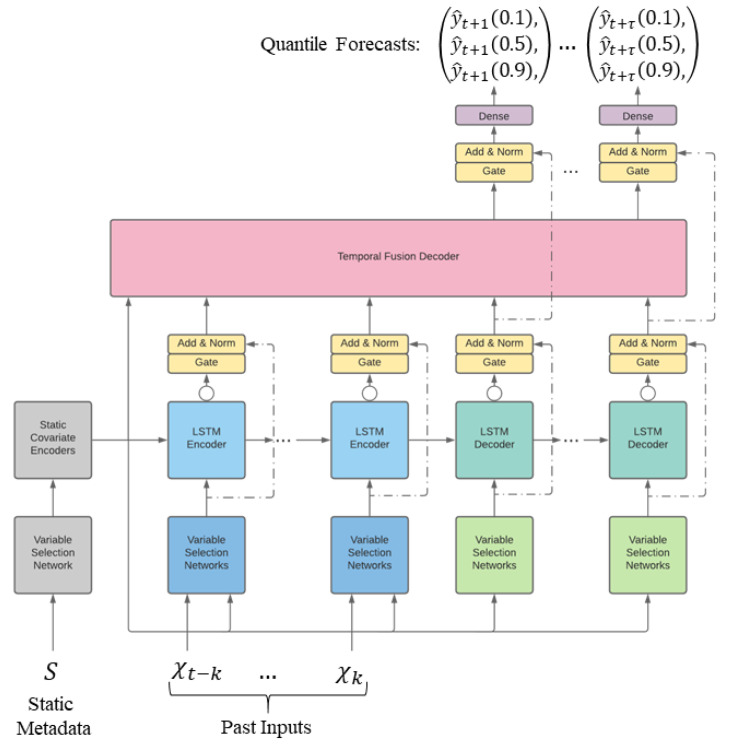
Temporal Fusion Transformers (TFT) architectural design. TFT inputs static attributes, time-varying historical inputs and time-varying a priori known future inputs. Variable filtering is used to allow a judicious selection of the most excellent features based on input. Gated Residual Network Blocks allow efficient information flow with skip and gating layer connections. Time-dependent processing is built on LSTMs for local processing and multi-head attention for the aggregation of information at any time.

Figure 12 indicates a high-level structure of TFT, with individual components described in detail in [31].

## 4. Experimental Results and Discussion

In this section, the experimental results are analyzed for each block of Figure 4. It is noteworthy to mention that the A2D2 dataset has not been used in the context of health diagnosis; hence, there are no benchmarks to directly compare the obtained detection results. However, the research by Biddle et al. [29] provides a good basis to compare results. The authors provided a fault detection, identification, isolation and prediction framework using support vector machine for autonomous vehicles. The same assumptions are made for the fault types and characteristics for detection and identification. The key difference is that [29] used simulation data and in this study real sensor measurements were used as a ground truth. I the following sections, we will compare our detection and isolation results with this study as it serves as the closest study that can resemble a baseline. The authors reported the accuracy of 94.94%, 97.42% and 97.01% for detection, isolation and identification, respectively.

For comparison of the forecasting strategy, [31] provides a comprehensive comparative study, demonstrating the performance of the TFT model with respect to other state-of-the-art approaches. Another challenge is that other well-known datasets for autonomous driving only provide limited information from vehicle bus data. For instance, KITTI [32] and ApolloScape [33] only provide GPS and IMU data and Waymo OD [34] includes velocity and angular velocity of the autonomous vehicle.

### 4.1. Sensor Fault Detection

As mentioned in Section 2, the dataset generated to train and test (data from Ingolstadt and Munich) the proposed fault detection system comprised of 20,097 signals (11,107 signals for training and 8990 signals for testing), with each signal containing the data-point amplitudes for each sensor of 2.4 s interval (equal to 240 data points). As described in Section 2, we have used the data from Ingolstadt, Munich and Gaimersheim for training, testing and validation, respectively. This has been set deliberately to ensure a good variety of training scenarios are captured, and our setup is open-set. This will help reduce the generalization error. To address the unbalanced amount of data (a more significant number of faulty signals compare to healthy ones), we have used oversampling strategy in our training split.

After the application of oversampling for training split of the data, a total of 10,720 signals is available for healthy scenarios and 10,724 signals for faulty scenarios. For testing, we have used 8990 signals, of which 310 signals are healthy, and 8680 signals are faulty.

**Figure 13:**
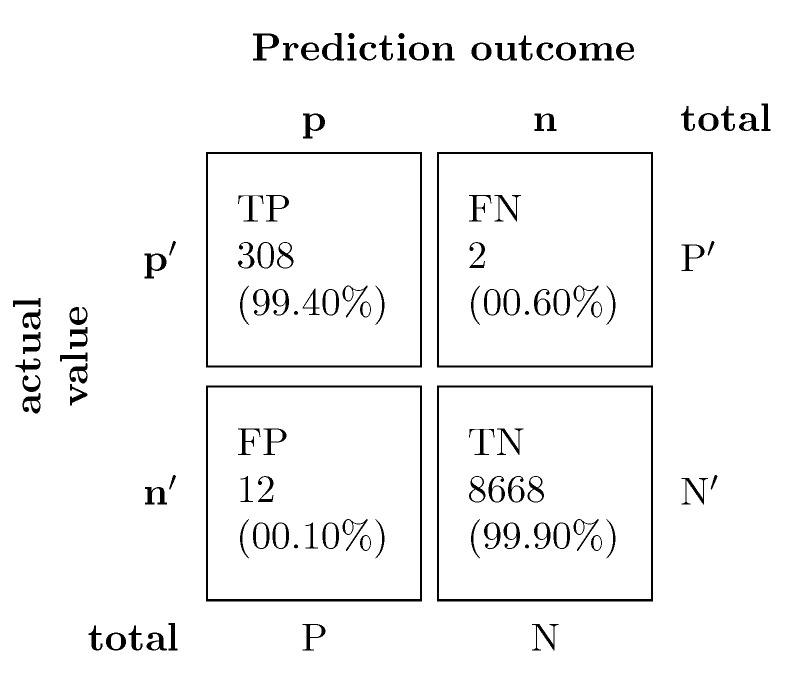
The confusion matrix of our detection system which achieved an accuracy of 99.84%.

Our CNN architecture achieves an accuracy of 99.84% for the detection task. For further analysis of detection results, we have presented the corresponding confusion matrix in Figure 13.

The confusion matrix shows that the detection system has almost no bias towards the prediction of healthy signals, indicating that the use of the oversampling technique will not result in an over-fitting issue.

Table 7 compares the sensor fault detection performance of the SVM and the proposed CNN frameworks. Since the A2D2 dataset is recently released, there is no comparable baseline performance in the literature for comparison. To create the baseline for our detection platform a SVM (famous in this field of research) classifier is deployed. Table 7 shows that the proposed CNN architecture outperforms the SVM baseline system.

### 4.2. Sensor Fault Identification and Isolation

SVM and DNN models are trained on the same training dataset used in the detection stage, while non-faulty signals are labeled 0. The results of both models’ for signal isolation have been shown in Table 8 which demonstrates that the deep learning technique outperforms SVM for all three sensors.

Identification accuracies are tabulated in Table 9. The identification performances vary depending on which sensor is evaluated. This is demonstrated in the findings of multi-class DNN, with a minimum average accuracy of 94.66%. Identification accuracy ranges from 81.00% to 100.00%, with the highest accuracy for brake pressure. As it can be seen, all fault types except drift fault are identified by accuracy of more than 85.00%. In this case, the model was able to detect the faults as an anomaly sample successfully. The highest accuracy has been achieved for the brake pressure sensor where the signal has the lowest fluctuation, and faults can be identified with an average accuracy of 98.00%.

### 4.3. Sensor Health Forecasting

Same as detection, isolation and identification for testing our prediction network, we have split the data to convert our problem to the open-set scenario, which is harder to predict and more generalizable. For this aim, we have used all available HI measures from Ingolstadt and all signal combinations (combinations described in Table 4) for training the TFT network and keep the HI measures from Munich for testing.

The TFT network takes a three-dimensional input with this format: [Time from the start, categorical ID (which is the city’s name), HI samples]. For the electronic sensor fault prediction problem, the look-back is set to 100 HI data-points and the look-forward is set to 50 HI data-points. It means that the model makes predictions based on the last 100 HI data-points, which are equal to 240 s (In the first iteration of the for-loop, the input carries the first 240 s, and the output is HI prediction for the next 120 s). It is worth mentioning that the look-back and look-forward sizes could be extended to higher values using more powerful processing units.

Our TFT network has an internal state size (size of the hidden layer) of 40 across the network with a learning rate of 0.001. To make the TFT model robust to changes, the dropout function is used. Dropout randomly drops 40% of units from the network. To train data, 100 epochs and a mini batch-size of 64 are used.

#### 4.3.1. Performance Measure

In a given time series dataset, let there be *I* particular entities; such as different calculated HI time series (based on all faulty/healthy combinations) in different cities. Each *i* object is correlated with a collection of $s_{i}$ static covariates, as well as $X_{i,t}$ inputs and $y_{i,t}$ scalar targets at each *t* time-step.

The availability of forecast intervals can be helpful in many situations to improve decisions and risk assessment by providing an indicator of the possible best and worst-case values that can be taken by the target. As such, we used quantile regression in this research work to set multi-horizon forecasting settings (e.g., outputting the 10th, 50th and 90th percentiles at each time step). As it is shown in Figure 12, each quantile forecast takes the form:

(1) $${\hat{y}}_{i}\left(q,t,\tau \right)=f_{q}\left(\tau ,y_{i,t-k:t},x_{i,t-k:t+\tau },s_{i}\right)$$

where ${\hat{y}}_{i,t+\tau }\left(q,t,\tau \right)$ is the forecasted *q*th sample quantile of the τ-step-ahead forecast at time *t*, and $f_{q}\left(.\right)$ is a forecast model. Similar to other direct methods, $\tau_{max}$ forecasts are outputted simultaneously. We insert all past knowledge into a finite look-back window *k*, using goal and known inputs only up till and including the forecast start timestamp *t* and known inputs over the full range.

TFT is trained by jointly minimizing the quantile loss, summed through all quantile outputs [35]. For this research, by concentrating on P10, P50 and P90 risk, we calculate the normalized quantile losses over the entire forecasting horizon.

#### 4.3.2. Prediction Results

The TFT model is trained using the HI time series from the combinational scenarios (as it is tabulated in Table 4) of Ingolstadt data using three different degradation paths. The number of data-points in the HI time series used for training, validation, and testing the TFT model are shown in Table 10. Table 11 shows the quantile loss for the test data; P10, P50 and P90.

**Figure 14:**
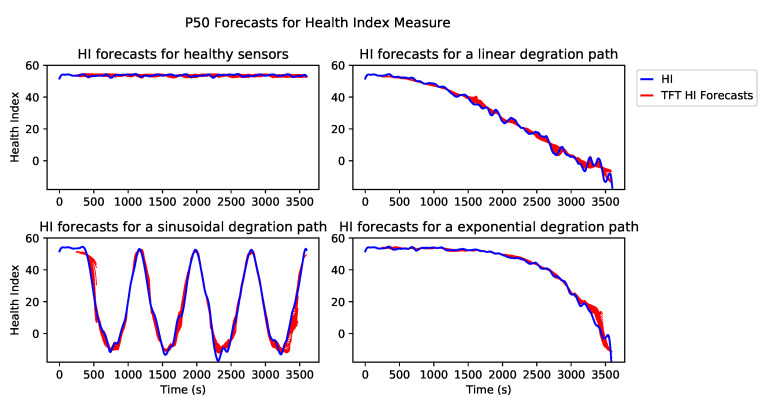
Test data vs. TFT predictions.

Figure 14 shows the plot of test data versus the TFT forecasts (using P50 quantile forecast) for three different degradation paths; linear, exponential and sinusoidal. The blue and red plots in Figure 14 show the original test HI time series and the forecasts generated by the TFT model, respectively. The forecasts are started after observing the first 100 HI data sequences, and at each step the model generates 50 future HI data sequences. We have plotted all forecasts generated at each step. These sequences have overlap, i.e., at the first step, TFT generates 50 future HI sequence by observing the first 100 HI data-points (0 to 99 HI data-points), and in the second step, it forecasts another batch of 50 HI data-points by observing the next overlapping analysis window of HI data-points which is from 1 to 100 HI data-points. This is why we have multiple overlapping forecasts per each HI data-points. In other words, the TFT forecasts consist of multiple predictions for moving horizon.

The top left plot of Figure 14 shows the original and forecasts of HI time series for the scenario in which all three sensors are healthy. The top right plot shows the original and forecasts of the HI time series for the system, which has a spike fault with a linear degradation path on the AccP sensor and healthy BP and SWA sensors. The bottom left plot shows an original and forecasts of the HI time series for the system, which has an erratic fault with a sinusoidal degradation path on AccP, BP sensors and healthy SWA sensor. The bottom right plot shows an original and forecasts of the HI time series for the system, which has a drift fault with an exponential degradation path on all three sensors: AccP, BP and SWA.

**Figure 15:**
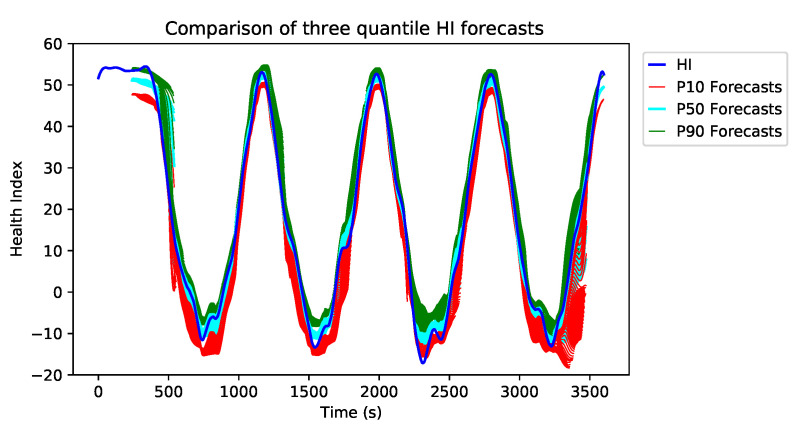
Test data vs. TFT forecasts for three different quantiles.

Figure 15 illustrates the comparison of three different quantile forecasts. Quantile forecasts are generated using linear transformation of the output from the temporal fusion decoder.

The example in Figure 15 shows a linearly degraded BP sensor with erratic fault and three sets of forecasts, which are P10, P50 and P90. Each of these forecasts aims to predict the low (10% quantile), mean (50% quantile) and the upper (90% quantile) ranges of HI. This implies that the P10 forecasts are much more conservative and tend to predict the worst-case scenario. On the other hand, the P90 forecasts tend to generate naive predictions. P50 forecasts are the closest approximation to the original HI time series. It is evident from Figure 15 that the P10 forecasts (red plot) are the most pessimistic approximation of the original HI stream (blue plot). The use of different quantile predictions is purely based on the application, i.e., for sensitive applications, the P10 approximation is more suitable since it predicts the worst-case scenarios.

## 5. Conclusions

This paper has examined the physical underpinnings of sensor faults and has mapped them to four main categories. The ultimate goal has been to enable a vehicle sensors health monitoring framework consisting of fault detection, isolation, identification and prediction. In that spirit, a CNN-based sensor fault detector/classifier has been shown to effectively manage a series of faults understudy for a large variety of fault combinations that occurred in a multi-sensor (in our case three sensor output) scenario. The proposed fault detection system obtained an accuracy of 99.84%. Based on the outcome of the fault detection, the next stage will be chosen; if the fault is identified, the signal will pass to the isolation and identification systems, and if no fault is detected, it will be passed to the sensor health forecasting system.

It has been shown that the proposed architecture, with deep learning algorithms, has the ability for autonomous vehicles to perform fault isolation and identification tasks. Key findings on the implementation of DNN models in multi-sensor control systems revealed high isolation and identification accuracy for different sensors when models are exposed to multiple fault types, such as drift fault, which is the most difficult to recognize. The multi-class DNN fault identification system obtained various performances for different fault types and sensors from 73.00% to 100.00%.

When the fault detection system shows no fault in the sensors, then the signals will pass to the sensor health forecasting system. In order to forecast the health state of the sensors, the output of the sensor fault detection system has used to define a HI measure by using three different degradation paths. The HI measure is the input to the TFT network to predict the sensors’ future behavior and forecast the possible faults before they occur. The performance measure which is used in this research is the quantile loss and the results have been reported for P10, P50 and P90 quantiles, with an obtained loss of 0.0315, 0.0611 and 0.0299, respectively. The proposed method is validated on the real-world dataset, A2D2 which is recently released by Audi (a German car manufacturer) [21].

## Figures and Tables

**Table 1:** Overview of machine learning and deep learning approaches for health management systems [11].

Method	Strengths	Limitations
**Machine Learning**		
Decision Trees (DT)	Easy to understand Non-parametric Good visualization	Requires prior knowledge May over fit Local minima problem
Random Forest	Improved performance to DT Fast and scalable Generally trains faster than SVM	Increased bias
Support Vector Machines	Fewer parameters to optimize Robust against noise Efficient for large datasets	Requires prior knowledge Incomprehensible results Memory intensive Designing the kernel function
Hidden Markov Model	Statistical Models Scalable	Requires prior knowledge Can become complex Large amounts of data is needed
**Deep Learning**		
Autoencoders	Can be equipped with CNNs to learn richer representations Easy to implement Dimensionality reduction Easy to track the loss function	Training requires a lot of data, Processing time and fine tuning Learns to capture as much information as possible rather than relevant information
Denoising Autoencoders	Better for compression/feature extraction Implicitly designed to form a generative model	Randomly inserts noise at input level
Variational Autoencoders	Learns to insert noise distributions Explicitly designed to form a generative model	Can be difficult to optimize Can be difficult to implement
Restricted Boltzmann Machine	Generative models Can create patterns if there are missing data	Can be difficult to train Difficult to track the loss/cost function
Deep Boltzmann Machine	Can learn good generative models Incorporates uncertainty about ambiguous inputs	Impractical for large datasets Approximate inference is slow
Deep Belief Networks	Good for 1D data Can extract global features Steady high performance on signals A powerful alternative to PCA	Training can be very slow and inefficient
CNN	Good for multi-dimensional data Extracts local features well	Might require more training
RNN, LSTM, and GRU	Good for sequential data Can detect changes over time	Can be difficult to train and implement

**Table 2:** Definitions of sensor outliers with its corresponding references [16].

Ref.	Definitions
[17]	”An outlier is an observation that digress significantly from others as to raise concerns that a different process created it.”
[18]	”An outlier is a data point that is substantially distinct from one another. From other data points, or is not compatible with the data points Anticipated typical behavior or corresponds well to a given normal behavior Anomalous conduct.”
[19]	”As a spatial-temporal outlier, a spatial-temporal point whose non-spatial class labels vary considerably from those of other temporally and spatially referred points in its spatial as well as temporal surroundings is considered.”
[20]	”An outlier is an observation or segment of occurrences which tends to be incompatible with the remaining dataset.”

**Table 3:** Available A2D2 data-points from AccP, SWA and BP sensors for each recording location.

Location	AccP	SWA	BP
Gaimersheim	52,576	52,577	52,576
Ingolstadt	91,968	91,968	91,969
Munich	74,520	74,520	74,521
Total	219,064	219,065	219,066

**Table 4:** Number of signals in all of the faulty/healthy combinations.

Channels	Healthy	Single Fault			Double Fault			Triple Fault
AccP	H	F	H	H	F	F	H	F
SWA	H	H	F	H	F	H	F	F
BP	H	H	H	F	H	F	F	F
Total	912	10,944			10,944			3648

**Table 5:** Fault parameters. * *SL* stands for signal length and ** *Sig* stands for signal.

Fault	Amplitude	Gradient	Max Length
Drift	Maximum *FSO*	Max $\pm \frac{FSO}{SL}$	$\frac{SL}{4}$
Hard-over	$1.1\times Sig^{**}FSO$	N/A	$0.5\times SL^{*}$
Erratic	±0.2×SigFSO	N/A	0.4×SL
Spike	0.5×SigFSO	N/A	N/A

**Table 6:** Time-domain feature definitions used for *N* data points in a sample *X*.

$f_{RMS}=\sqrt{\frac{1}{N}\sum_{i=1}^{N}x_{i}^{2}}$	$f_{SRA}={\left(\frac{1}{N}\sum_{i=1}^{N}\sqrt{|x_{i}|}\right)}^{2}$
$f_{KV}=\frac{1}{N}\sum_{i=1}^{N}{\left(\frac{x_{i}-\mu_{i}}{\sigma }\right)}^{4}$	$f_{SV}=\sum_{i=1}^{N}{\left(\frac{x_{i}-\mu_{i}}{\sigma }\right)}^{3}$
$f_{PPV}=max\left(x\right)-min\left(x\right)$	$f_{CF}=\frac{max(|x|)}{f_{RMS}}$
$f_{IF}=\frac{max(|x|)}{\frac{1}{N}\sum_{i=1}^{N}\left|x\right|}$	$f_{MF}=\frac{max(|x|)}{f_{SRA}}$
$f_{SF}=\frac{max(|x|)}{\sqrt{\frac{1}{N}\sum_{i=1}^{N}x_{i}^{2}}}$	$\frac{\frac{1}{N}\sum_{i=1}^{N}{\left(\frac{x_{i}-\mu_{i}}{\sigma }\right)}^{4}}{{\left(\frac{1}{N}\sum_{i=1}^{N}x_{i}^{2}\right)}^{2}}$

**Table 7:** Sensor fault detection performance compared to a baseline.

Method	Accuracy	AUC	F1 Score	Precision	Recall
SVM	98.55%	0.9441	75.47%	62.50%	95.24%
Proposed CNN	99.84%	0.9992	**97.78%**	**99.35%**	96.25%

**Table 8:** Sensor isolation accuracy for SVM and DNN.

Signal	SVM	DNN
Brake pressure	85%	97%
Accelerator pedal	81%	94%
Steering angle	83%	96%

**Table 9:** Fault identification accuracy by multi-class DNNs.

Signal	Erratic	Drift	Hard-Over	Spike	Overall
Brake pressure	100%	82%	100%	100%	98%
Accelerator pedal	85%	73%	100%	100%	89%
Steering angle	100%	80%	100%	100%	97%

**Table 10:** Number of data-points in HI time series for train, validation and test of the TFT model. *path. stands for degradation path.

	Sensor Condition									
	**Healthy**	**Erratic Fault**			**Spike Fault**			**Drift Fault**		
path.*	**N/A**	**Lin.**	**Exp.**	**Sin.**	**Lin.**	**Exp.**	**Sin.**	**Lin.**	**Exp.**	**Total**
Train	1.5 k	33 k	33 k	33 k	33 k	33 k	33 k	33 k	33 k	265.5 k
Valid.	840	18.5 k	18.5 k	18.5 k	18.5 k	18.5 k	18.5 k	18.5 k	18.5 k	148.8 k
Test	1.2 k	26.4 k	26.4 k	26.4 k	26.4 k	26.4 k	26.4 k	26.4 k	26.4 k	212.4 k
Total	3.5 k	233.7 k			233.7 k			155.8 k		626.7 k

**Table 11:** Quantile loss for test data.

P10	P50	P90
0.0315	0.0611	0.0299

## Data Availability

Publicly available datasets were analyzed in this study. This data can be found here: [https://github.com/MAminSFV/multi-sensor-FDII-health-forecasting-for-autonomous-vehicles] (accessed on 1 January 2021).

## Abbreviations

The following abbreviations are used in this manuscript:

MDPI	Multidisciplinary Digital Publishing Institute
DOAJ	Directory of open access journals
TLA	Three letter acronym
LD	Linear dichroism
PdM	Predictive maintenance
SVMs	Support vector machines
K-NN	K-nearest neighbor
ANNs	Artificial neural networks
DNNs	Deep neural networks
RNN	Recurrent neural networks
CNN	Convolutional neural networks
RUL	Remaining useful life
HI	Health index
TFT	Temporal fusion transformers
A2D2	Autonomous driving dataset
AccP	Accelerator pedal
SWA	Steering wheel angle
BP	Brake pressure
FSO	Full-scale output
LSTM	Long short-term memory
